# A Descriptive Quantitative Analysis on the Extent of Polypharmacy in Recipients of Ontario Primary Care Team Pharmacist-Led Medication Reviews

**DOI:** 10.3390/pharmacy8030110

**Published:** 2020-06-30

**Authors:** Nichelle Benny Gerard, Annalise Mathers, Christoph Laeer, Eric Lui, Tom Kontio, Payal Patel, Lisa Dolovich

**Affiliations:** 1 Leslie Dan Faculty of Pharmacy, University of Toronto, 144 College St, Toronto, ON M5S 3M2, Canada; nichelle.bennygerard@mail.utoronto.ca (N.B.G.); annalise.mathers@utoronto.ca (A.M.); 2 Family First Health Centre, 4270 Innes Rd, Orléans, ON K4A 5E6, Canada; christoph.laeer@ffhc.ca; 3 North York Family Health Team, 707-240 Duncan Mill Road, Toronto, ON M3B 3S6, Canada; elui@nyfht.com; 4 Thames Valley Family Health Team, 6-1385 N Routledge Park, London, ON N6H 5N5, Canada; tom.kontio@thamesvalleyfht.ca (T.K.); Payal.patel@thamesvalleyfht.ca (P.P.); 5 Department of Family Medicine and Health Evidence and Impact, McMaster University, 100 Main St W, Hamilton, ON L8P 1H6, Canada

**Keywords:** polypharmacy, morbidity, potentially inappropriate medications (PIMs), primary care, pharmacists, medication reviews, drug therapy management

## Abstract

Pharmacist-led medication reviews have been shown to improve medication management, reducing the adverse effects of polypharmacy among older adults. This paper quantitatively examines the medications, medication discrepancies and drug therapy problems of recipients in primary care. A convenience sample of 16 primary care team pharmacists in Ontario, Canada contributed data for patients with whom they conducted a medication review over a prior four-week period. Data were uploaded using electronic data capture forms and descriptive analyses were completed. Two hundred and thirty-seven patients (on average, 67.9 years old) were included in the study, taking an average of 9.2 prescription medications (±4.7). Majority of these patients (83.5%) were categorized as polypharmacy patients taking at least five or more prescribed drugs per day. Just over half of the patients were classified as having a low level of medication complexity (52.3%). Pharmacists identified 2.1 medication discrepancies (±3.9) and 3.6 drug therapy problems per patient (±2.8). Half these patients had more than one medication discrepancy and almost every patient had a drug therapy problem identified. Medication reviews conducted by pharmacists in primary care teams minimized medication discrepancies and addressed drug therapy problems to improve medication management and reduce adverse events that may result from polypharmacy.

## 1. Introduction

Chronic diseases such as diabetes, hypertension and heart disease are the leading cause of death worldwide and pose a significant challenge for healthcare providers to address in a comprehensive and coordinated manner [1,2,3]. The World Health Organization projects that the deaths due to chronic diseases will increase from 38 million in 2012 to 52 million by 2030 [2]. Moreover, approximately one in three Canadians live with at least one major chronic disease and this is expected to rise as a result of an aging population and other lifestyle risk factors [1,4]. Care for such patients is a growing focus in health services research and public health policy [1].

Multimorbidity, the co-occurrence of two or more chronic diseases, adds another layer of complexity to the therapeutic management of chronic diseases [5,6]. Navickas et al. report that 95% of the primary care population aged 65 years and older are afflicted with multimorbidity [7]. There is considerable strain on healthcare systems as multimorbidity is increasingly recognized as a cause of poor health outcomes, increased health service use, and associated costs [5,8,9]. Age is a risk factor in developing chronic diseases and patients with multiple diseases are at a greater risk of adverse health outcomes, more frequent hospital admissions, longer hospital stays, regular medical specialist visits, and mortality [6,10,11].

Patients with multimorbidity often require multiple drugs to achieve optimal clinical management [12,13]. The use of more medications than clinically indicated or the use of five or more prescribed drugs per day is referred to as polypharmacy [14]. Globally, the elderly population takes an average of two to nine medications per day [14]. Polypharmacy is an area of particular concern for medication management since the prevalence of inappropriate medication usage by the elderly is reported to vary between 11.5% and 62.5% [15]. With increasing numbers of medications, the risk of adverse outcomes from drug–drug and drug–disease interactions simultaneously increases, leading to adverse drug reactions, medication non-adherence, reduced physical capacity, frequent hospital visits and mortality [11,16,17]. These events are further exacerbated due to the metabolic changes and reduced drug clearance associated with aging [18]. Polypharmacy is therefore known to be common among the older adult population who experience multimorbidity, and so identifying and reducing the number of medications a patient is receiving can lead to better outcomes and help improve quality of life [11,19].

Although healthcare services and guidelines are available for the management of multimorbidity, past research has found that these services are rarely adequately designed to meet the clinical challenges of treating multiple chronic diseases and are primarily developed based on trials of interventions for single diseases [7,9,20]. Consequently, older patients often receive care that is complex, inefficient, ineffective and fragmented [21]. There are several interventions that attempt to reduce potentially inappropriate medication usage to mitigate risks associated with multimorbidity, however, these require collaboration between various health care providers so that a complete picture of a patient’s medical condition is understood and addressed comprehensively by the healthcare team [17,18]. Spinewine et al. suggest that primary care must move towards a team-based care approach consisting of patients, the patient’s primary care provider and other health care professionals in order to improve clinical decision making, collaboration and communication, adherence, and monitoring [20].

Pharmacists, in particular, are increasingly becoming integrated into interdisciplinary primary health care teams. In Canada, the province of Ontario has over 170 pharmacists integrated into primary care team settings including Family Health Teams (FHTs, or interdisciplinary group practices) and Community Health Centres (CHCs) [22]. Within these teams, pharmacists engage in many direct patient care activities including medication management, identifying adverse medication usage, and patient education, notably through the delivery of medication reviews [23,24]. When working in a primary care team setting, pharmacists are typically paid a salary and as such are not remunerated on a fee-for-service basis for each medication review or other professional service activities [25,26]. Ontario pharmacists do not have broad prescribing authority although they are able to initiate therapy for smoking cessation. Pharmacists are able to renew or adapt a prescription if they have the original prescription order, unless it is for a controlled substance. They cannot order or interpret lab tests [25,26].

Pharmacist-led medication reviews in primary care have been shown to improve management of chronic disease and help to avoid adverse effects that result from polypharmacy [3]. Medication reviews are also known to decrease the number of drug therapy problems and inappropriate medications by altering drug dosage, formulation and regimen [24].

The objectives of this study are to understand the extent of polypharmacy, the processes and selected consequences of pharmacist-led medication reviews done in FHTs in the province of Ontario, Canada. The study aims to answer the following:

1. What are the characteristics of patients on ≥3 chronic or concurrent medications who received a medication review conducted by Ontario primary care team pharmacists?
2. What is the average number of medications per patient visiting a pharmacist in primary care?
3. What proportion of patients that have been prescribed ≥3 chronic or concurrent medications have been identified with (a) DTPs and (b) medications discrepancies by Ontario primary care team pharmacists?

## 2. Materials and Methods

### 2.1. Study Design

This study was an observational retrospective chart review. Data were extracted from electronic medical records of patients who received a medication review service from a participating primary care team network pharmacist. The study was approved by the University of Toronto Research Ethics Board (application #37126, approved 14 February 2019) as well as Hamilton Integrated REB (HiREB) (approved 2 June 2019, Project Number: 7205) and OHRI/TOH OHRI Institutional Approval for Ottawa Health Science Network Research Ethics Board (OHSN-REB) Submission (approved 15 October 2019, Protocol ID#: 20190540-01H).

### 2.2. Study Sample

Pharmacists working at an Ontario primary care team site were invited to contribute data for all patients with whom they conducted a medication review in person or via phone over a prior four-week period. Pharmacists from different types of primary care team sites including primary care community sites (n = 10), 2 primary care sites located in an academic or hospital site (n = 2) and primary care sites adjacent to, but not officially governed by a hospital or academic site (n = 1), were invited to participate. Eligible patients included those who were taking ≥3 chronic or concurrent medications who received a medication review (excluding interactions limited only to medication reconciliation). There were no formal exclusion criteria. Patients seen by the pharmacist for other types of consultations, including brief follow-up reviews focusing on a single clinical issue, were not eligible for participation.

### 2.3. Intervention Studied and Justification

Participating pharmacists performed medication reviews with primary care team patients, in order to help patients manage their medications, as part of their normal everyday practice. The purpose of a medication review is to (1) ensure a patient’s medications are accurate and safe through medication reconciliation, as well as assessing patient’s medication and factors related to this including medication costs and insurance coverage, (2) focus on technical issues of a patient’s prescriptions such as discrepancies or out-of-date products, (3) identification and rectification of issues related to the use of medications, (4) education and encouragement of patients on the correct use of medications, and (5) the optimization of the appropriate, safe and effective use of medications [27,28]. The pharmacist performed a medication review in their primary care team following usual standards of practice including preparation for the review by examining patient records at the practice site, conducting a patient interview, and documenting the review in the patient record. No standard forms or templates were used during the conduct of the medication review.

### 2.4. Recruitment and Informed Consent

Primary care team pharmacists were recruited using a convenience sample through information about the study disseminated to the Ontario Primary Care Team Pharmacists Network [29]. There are approximately 200 primary care sites that pharmacists work at across the province of Ontario [29]. Pharmacists who consented to participate provided their medication review data. A waiver of consent for patients was obtained from the University of Toronto Research Ethics Board, Hamilton Integrated REB and OHRI due to the retrospective nature of the chart review. The pharmacist assigned each patient a unique identifier, which was not shared with the research team. No identifying patient information was uploaded, and all data uploaded was de-identified.

### 2.5. Data Collection and Management

Pharmacists uploaded patient medication review data using electronic data capture forms onto a secure, de-identified online database (REDCap) [30]. Data were collected using a structured data collection form that included select patient demographics, information about the processes and outcomes of conducting medication reviews. Each participating pharmacist was assigned a de-identified Site ID by the research team. Pharmacists entered de-identified patient data into RedCAP after the medication review was completed.

Data were collected and managed according to the following definitions and categorizations:

- Polypharmacy was defined as the use of five or more prescribed drugs per day [15].
- Patient complexity was categorized into three complexity levels as described in Appendix A, Table A1 [31]. A higher assigned level of complexity represents a patient with greater health and pharmaceutical complexity.
- DTPs were broadly defined as ‘an actual or potential undesirable event experienced by a patient which involves, or is suspected to involve, drug therapy and that interferes with achieving the desired goals of therapy’ classified according to the codes in Appendix A, Table A2 [32,33].
- Medication discrepancies were broadly defined as ‘any preventable event that may cause or lead to potentially inappropriate medication (PIM) use or patient harm while the medication is in control of the healthcare provider, patient or consumer’ [34]. The discrepancies in the data capture forms asked the pharmacist to classify the discrepancy as drug name, drug dose, drug frequency, or other.
- Pharmacists classified medications into general categories (ex. Medications for diabetes, medications for cardiovascular disease, etc.) based on their professional judgement and experience.
- Pharmacists noted discrepancies between the electronic medical record (EMR), and information identified during pharmacist medication review.

### 2.6. Data Analysis

Descriptive analyses were performed. Means and standard deviations were generated for continuous data. Proportions were generated for categorical data. Data were summarized in graphical and numerical form for review by the research team to identify patterns and assist with interpretation of the data. An attempt was made to clarify and resolve any questions regarding the number of DTPs recorded, number of medication discrepancies, number of medications, and participant characteristics with the pharmacist that collected the data.

## 3. Results

### 3.1. Patient Characteristics

In total, 16 pharmacists participated in the study working at 13 sites in total. There were 237 patients included in the study. The mean age of patients was 67.9 years (±13.9) and 54.8% were women (Table 1).

### 3.2. Types of Medications, Polypharmacy and Patient Complexity

As indicated in Table 2, patients took an average of 9.2 prescription medications (±4.7), 2.1 over-the-counter medications (±2.3), 0.4 natural or herbal products (±0.9), and 0.1 other medications (±0.8). The number and proportion of patients who were categorized as polypharmacy patients were n = 198 (83.5%).

Medications that were recorded included medications for diabetes, chronic pain, mental health conditions, cardiovascular conditions, respiratory conditions, osteoporosis, and for active cancer treatment. The majority of the patients were taking medications for hypertension (64.1%), medications for diabetes excluding insulin (38.8%), medications for chronic pain excluding opioids (35.8%), antidepressants (27.8%), sedatives (24.9%), opioids (24.5%) and insulin (19.0%) as shown in Figures S1–S17.

**Figure 1:**
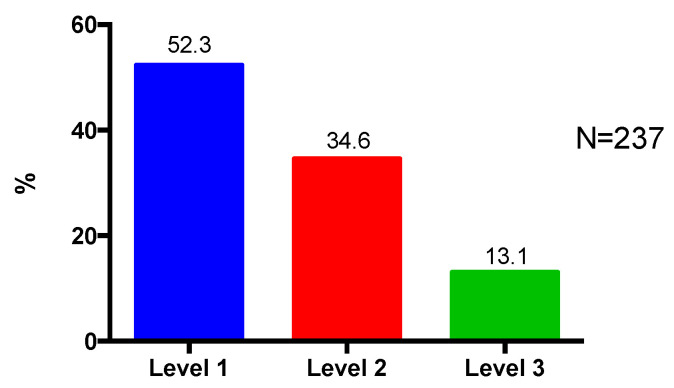
Patient complexity level.

Majority of the patients (52.3%) were classified by participating pharmacists as having Level 1 complexity (Figure 1).

### 3.3. Number of Medication Discrepancies

**Figure 2:**
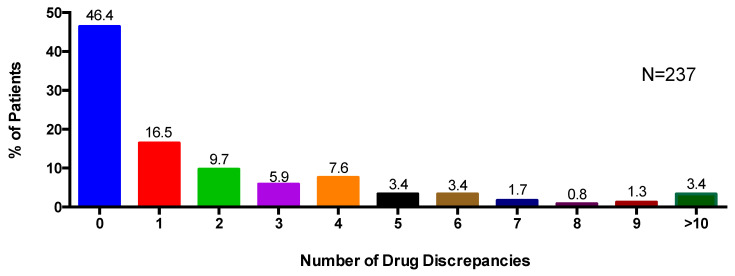
Percentage of patients with any number of drug discrepancies including drug name, drug dose, drug frequency, and other.

Pharmacists identified 500 discrepancies between the EMR and pharmacist medication review in the total patient sample. Patients had an average of 2.1 medication discrepancies (±3.9). Overall, just over half of the patients (53.6%) were identified as having at least one type of medication discrepancy (Figure 2). One patient had 39 medication discrepancies (the highest number) identified. The majority of medication discrepancies (55%) were related to drug name with 31.2% of the patients identified as having at least one drug name discrepancy (Table 3). Of the total discrepancies identified, 19% were related to drug dose; 11.4% were drug frequency discrepancies; and 14.6% were “other” discrepancies.

### 3.4. Number of Drug Therapy Problems

**Figure 3:**
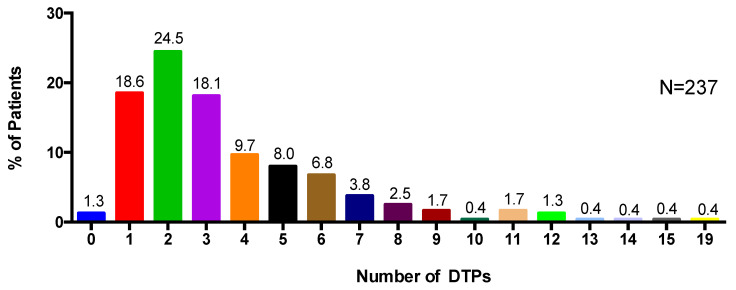
Percentage of patients and the number of DTPs identified.

In total, 860 drug therapy problems (DTPs) were identified by the primary care team pharmacists, with an average of 3.6 DTPs identified per patient (±2.8) (Table 4). The majority of DTPs (22.6%) identified were related to patients requiring drug therapy for an indication that the patient was not currently receiving or taking (Type 2 DTP). The percentage of patients with at least one Type 2 DTP out of the sample was 52.3% (Table 5). A small percentage of DTPs (3.4%) were classified as DTPs related to patients experiencing or having the potential to experience a drug–drug, drug–food, or drug–laboratory interaction (Type 8 DTP). Overall, almost all patients (98.7%) were identified as having at least one type of DTP (Figure 3) by participating pharmacists.

## 4. Discussion

The occurrence of polypharmacy in aging populations is well known as older patients often require multiple medications to treat their chronic conditions [35]. This study, which describes medication reviews conducted by pharmacists as part of interdisciplinary primary care teams, found that a large majority of patients cared for by the pharmacists were older adults classified as polypharmacy patients. Many of these patients were on medications that were difficult to manage or associated with high risk such as antidepressants, sedatives, opioids and insulin [36]. The 2019 AGS Beers Criteria^®^ for PIM Use in Older Adults is a widely used, explicit list of medications that should often be avoided for older adults, which includes many of the types of medications that this sample of patients reported using as part of their daily medication regimens [37]. These medications within the context of polypharmacy require a judicious balance of expected benefits and risks of adverse events when taken by older adults [36,37,38]. The data from this study provide further support that primary care team pharmacists are well placed to identify opportunities that improve medication safety and effectiveness especially for community based older adults taking PIMs that may lead to an increased risk of adverse events [35].

Despite many pharmacists classifying the patients as having a low complexity rating (i.e., the task is clear and well defined and all patient-related factors are present and easily interpreted), more than half of the patients had one or more medication discrepancies identified and majority of patients had at least one DTP. The complexity ratings may also have been categorized as low because the participating pharmacists were a very experienced group of pharmacists who were accustomed to dealing with complexity and as such could more easily handle cases that less experienced pharmacists may have rated as more complex.

In this study alone, 500 medication discrepancies were identified by pharmacists in the total patient sample and over half were drug name discrepancies. Research shows that medication-related errors and discrepancies account for approximately 44,000 to 98,000 fatalities per year and contribute to more deaths than breast cancer or HIV-related complications [39]. Older patients, such as those in our study, are at especially high risk of such medication errors because of polypharmacy leading to complicated medication regimens and inadequacy within the current medication information sharing system, which can exacerbate the effects of polypharmacy [40]. Studies indicate an exponential increase in the incidence of adverse drug reactions observed with initiating additional drug therapies to a patient’s regimen [41,42]. Our study supports that patients who have a high medication burden may experience an increased risk of medication errors, along with the increased risk of drug therapy problems such as drug–drug interactions, non-adherence, and increased overall drug expenditures among other things [43,44,45]. Pharmacists can play an essential role in identifying these errors where the prescriber may have changed, added or omitted a medication for a patient.

Polypharmacy has also been associated with a higher risk of DTPs and the risk of hospitalization [44]. A study conducted by Viktil et al., that compared patients taking five or more drugs with those that took less than five drugs, identified that the number of DTPs per patient increased linearly with the number of drugs used [45]. A cross sectional study on DTPs identified during medication reviews deemed overtreatment as the most frequent DTP [46]. This is consistent with a study conducted by Abdin et al., who detected an average of 7.2 DTPs per patient with the most common DTP as ‘drug use without indication’ [23]. Primary care team pharmacists are in an advantageous position to have access to a patient’s longitudinal health record and regular interactions with the rest of the interprofessional health care team so as to identify care gaps, be it overtreatment or undertreatment, where medications may provide benefit for untreated health conditions. This study takes place in Ontario, Canada which has recently transitioned to a centralized health administrative structure led by Ontario Health, which will oversee a collection of Ontario Health Teams (OHTs) providing health care coverage across the province. Each OHT is expected to provide integrated care across health care sectors within a local community. As OHTs develop it will become increasingly important to have data available, such as the data from this study to inform decisions on allocation of health care provider resources [47].

Pharmacists play a significant role in the reduction of polypharmacy through the provision of medication reviews. Thompson et al. determined that pharmacists usually prescribe fewer medications than physicians, which supports the movement of integrating pharmacists into primary care in attempts to reduce potentially harmful polypharmacy [48]. Medication reviews ensure a patient’s medications are accurate and safe, engage patients in their own medication management, improve medication self-efficacy and adherence, and contribute to overall quality of life [27,28,49,50,51]. Primary care pharmacists are optimally positioned to contribute to improving medication management and have been shown to reduce emergency room visits and hospitalizations as well as increase prescribing appropriateness, particularly for polypharmacy patients [27,52,53].

Pharmacist-led medication reviews are essential to examine a patient’s medications and to assess what actions need to be taken to minimize and correct medication discrepancies and DTPs to maximize drug-related benefits [39]. The identification and resolution of DTPs is one of the most significant contributions a primary care pharmacist can make. This study is consistent with other studies showing a high level of DTP identification among pharmacists based in primary team-based care settings [54,55]. Research has also shown that primary care team pharmacists have a higher rate of recommendations made and implemented as a result of medication reviews than those conducted by non-primary care team pharmacists [56,57]. Overall, pharmacists’ interventions have been shown to reduce incorrect or unsafe use of PIMs, DTPs, and medication discrepancies and is further supported by the research in Ontario, Canada where pharmacists, patients and health care professionals have reported improved quality of primary care [58].

The strengths of this study include the diversity of the sites involved across the province and the multiple pharmacists that had participated. The pharmacists in our study practiced in a mix of urban and rural areas, although there was a greater concentration of pharmacists in urban areas who chose to participate. This is also the first report that combines data from of pharmacists participating in the Ontario Primary Care Team Pharmacists Network Limitations include the convenience sampling approach of pharmacists and data collection only taking place over a four-week period. This sampling approach only provides a cross-sectional perspective regarding polypharmacy among patients in primary care teams and therefore may not be generalizable to the general population. The retrospective nature of the data collection also limited the feasibility of obtaining follow up data for the majority of patients included in the study. Data may be skewed slightly towards a higher medication burden due to the inclusion criteria of patients with ≥3 chronic or concurrent medications.

## 5. Conclusions

This study describing medication reviews conducted by pharmacists working in interdisciplinary team-based settings found that the majority of patients cared for by the pharmacists were polypharmacy patients. Many of these patients were taking PIMs that may place them at increased risk for adverse events. This study provides further evidence that primary care team pharmacists are well placed to minimized medication discrepancies and addressed drug therapy problems to improve medication management and reduce adverse events that may result from polypharmacy. Future studies on the impact on patient’s clinical outcomes will help to further quantify the value of DTP identification and resolution by pharmacists working in primary team-based care settings.

## Figures and Tables

**Table 1:** Patient participant characteristics.

Characteristic	Mean (SD) N: % (N = 237)
Age (in years)	67.9 (13.9)
Female	54.8%
Number of Prescription Medications	9.2 (4.7)
History of MedsCheck in the past year	11.4%
History of Health Service Utilization, Past Month (ER visit or hospital discharge)	21%

**Table 2:** Number of medications.

	Mean	Standard Deviation	Max Value	Min Value
Prescription	9.2	4.7	25	3
Over the Counter	2.1	2.3	20	0
Natural or Herbal Products	0.4	0.9	7	0
Other	0.1	0.8	9	0

**Table 3:** Number of patients with each type of medication discrepancy.

Type of Discrepancy	Number of Patients with at least One Discrepancy Identified *	% (of Total Patients with at least One Discrepancy Identified)
Drug Name	74	31.2
Drug Dose	51	21.5
Drug Frequency	31	13.1
Other	20	8.4

* one patient may have more than one type of medication discrepancy.

**Table 4:** Number of drug therapy problems [30].

Type of DTPs		Number of DTPs Identified	% (of Total DTPs)
Type 1	Receiving/taking drug with no valid indication	114	13.3
Type 2	Requires drug therapy for an indication and is not receiving/taking therapy	194	22.6
Type 3	Not receiving/taking appropriate drug or drug product	121	14.1
Type 4	Receiving/taking too little drug	75	8.7
Type 5	Receiving/taking too much drug	93	10.8
Type 6	Not receiving/taking prescribed drugs appropriately	105	12.2
Type 7	Experiencing an adverse drug reaction (not dose-related)	91	10.6
Type 8	Experiencing a drug–drug, drug–food, or drug–laboratory reaction	29	3.4
Type 99 (Other) ^i^	Other	38	4.4
TOTAL		860	100

^i^ Examples of a Type 99 (Other) DTP can be seen in Appendix A, Table A2.

**Table 5:** Number of patients with each type of DTP.

Type of DTP	Number of Patients with at least One DTP Identified *	% (of Total Patients with at least One DTP Identified)
Type 1	65	27.4
Type 2	124	52.3
Type 3	60	25.3
Type 4	55	23.2
Type 5	66	27.9
Type 6	63	26.6
Type 7	61	25.7
Type 8	20	8.4
Other	26	11.0

* one patient may have more than one type of DTP.

## Supplementary Materials

The following are available online at https://www.mdpi.com/2226-4787/8/3/110/s1, Figure S1: Insulin, Figure S2: Other Medications for Diabetes, Figure S3: Opioid Medications, Figure S4: Other Medications for Chronic Pain, Figure S5: Antipsychotic Medications, Figure S6: Antidepressant Medications, Figure S7: Sedative drugs (e.g., benzodiazepines, zopiclone, etc.), Figure S8: Other Medications for Mental Health Conditions, Figure S9: Warfarin or DOACS, Figure S10: Hypertension, Figure S11: Congestive Heart Failure, Figure S12: Asthma, Figure S13: COPD, Figure S14: Osteoporosis, Figure S15: Fall in Previous Year, Figure S16: Active Cancer Treatment, Figure S17: Cancer Survivor.

## Appendix A. Definitions and Classifications

**Table A1:** Patient Complexity Level [29].

Complexity Level	Factors
Level 1—low	The task (e.g., medication assessment, focused drug question) is clear (well defined). All patient-related factors are present and easily interpreted.
Level 2—moderate	The task referred to the pharmacist is unclear or not well defined. The pharmacist has to collect initial information before clearly defining the task. The patient is taking multiple medications and has multiple medical conditions requiring drug therapy. The clinical knowledge/skill required to address the task is complex. The DTPs identified are complex.
Level 3—high	Both the case scenario and the clinical knowledge are complex and ill-defined and multiple DTPs that are codependent are present.

**Table A2:** DTP Classification [30].

Type of DTPs	Factors
Type 1	Receiving/taking drug with no valid indication
Type 2	Requires drug therapy for an indication and is not receiving/taking therapy
Type 3	Not receiving/taking appropriate drug or drug product
Type 4	Receiving/taking too little drug
Type 5	Receiving/taking too much drug
Type 6	Not receiving/taking prescribed drugs appropriately
Type 7	Experiencing an adverse drug reaction (not dose-related)
Type 8	Experiencing a drug–drug, drug–food, or drug–laboratory reaction
Type 99 Other	Other; examples include requesting further assessment from physician; laboratory parameters elevated or problematic and pharmacist recommends non-drug therapy; requesting laboratory monitoring to be completed
0	Unsure
10	Not a DTP
