# Supplementary material for: A Descriptive Quantitative Analysis on the Extent of Polypharmacy in Recipients of Ontario Primary Care Team Pharmacist-Led Medication Reviews
Source: Pharmacy (Basel). 2020 Jun 30;8(3):110. doi: 10.3390/pharmacy8030110 (PMC7558087; doi:10.3390/pharmacy8030110)
Supplement: Supplementary file 1 [file pharmacy-08-00110-s001.pdf]

Supplementary Materials

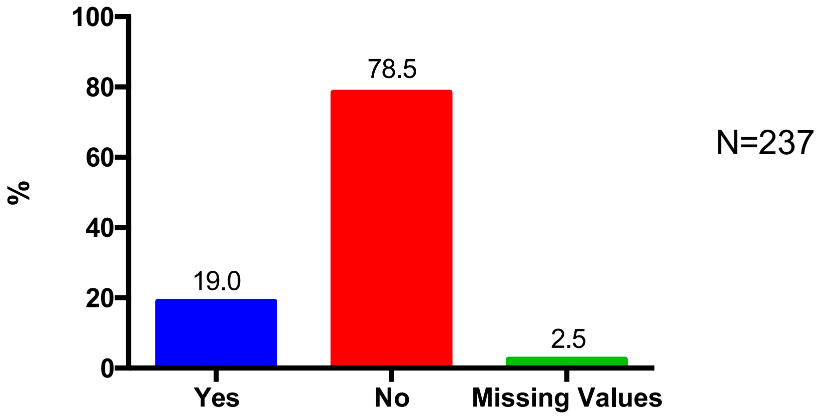

Figure S1. Insulin

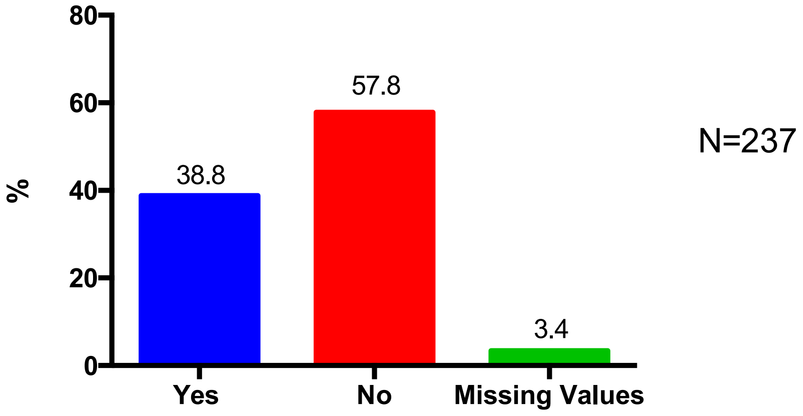

Figure S2. Other Medications for Diabetes

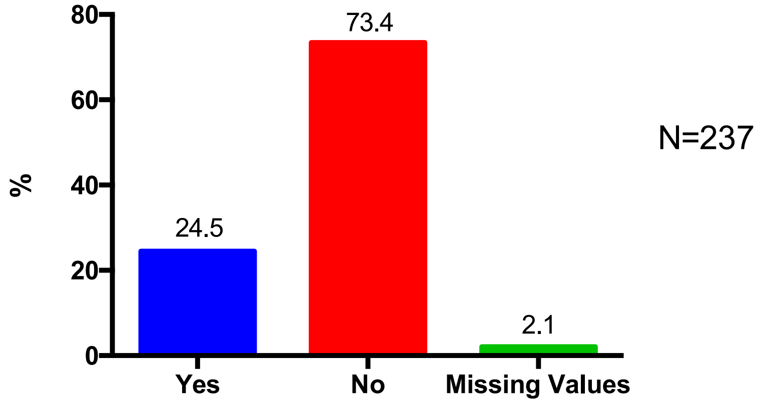

Figure S3. Opioid Medications

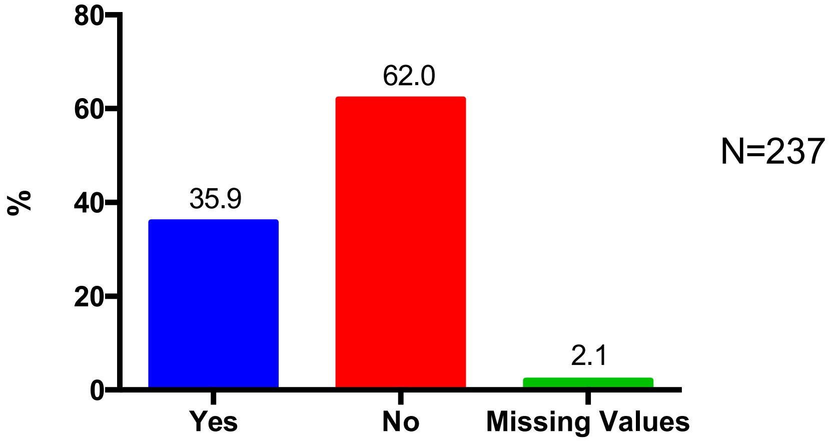

Figure S4. Other Medications for Chronic Pain

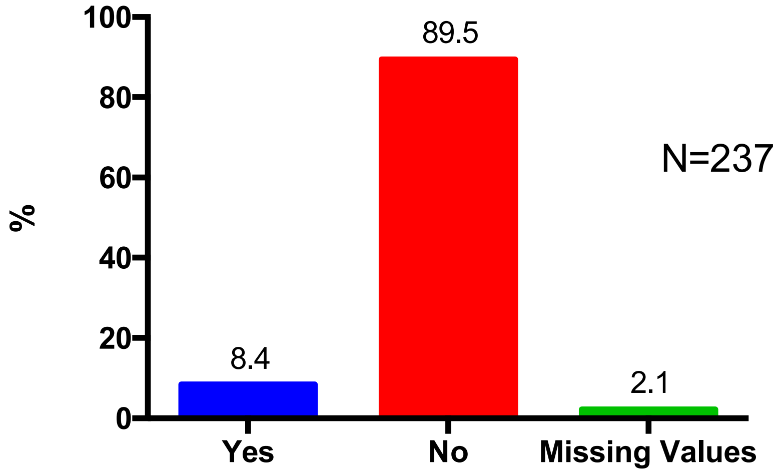

Figure S5. Antipsychotic Medications

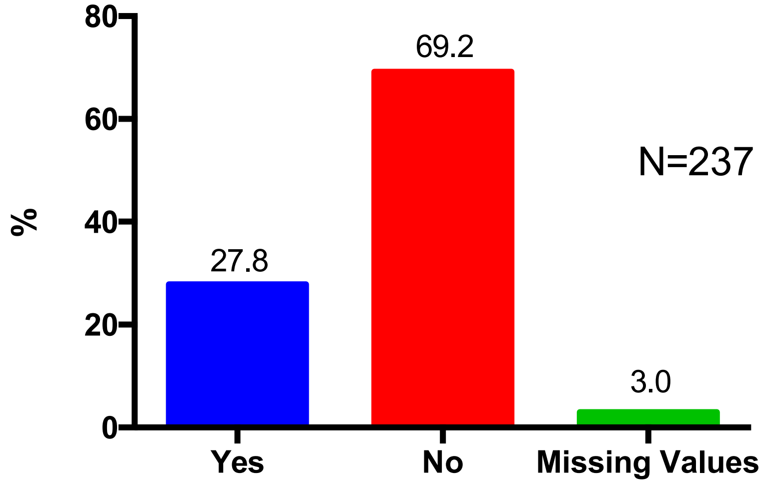

Figure S6. Antidepressant Medications

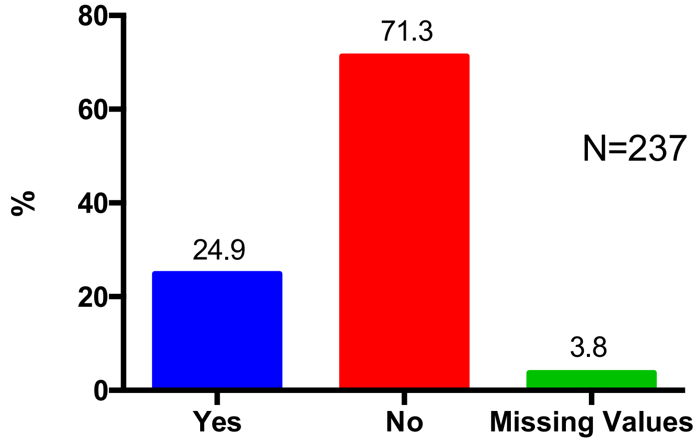

Figure S7. Sedative drugs (e.g. benzodiazepines, zopiclone, etc.)

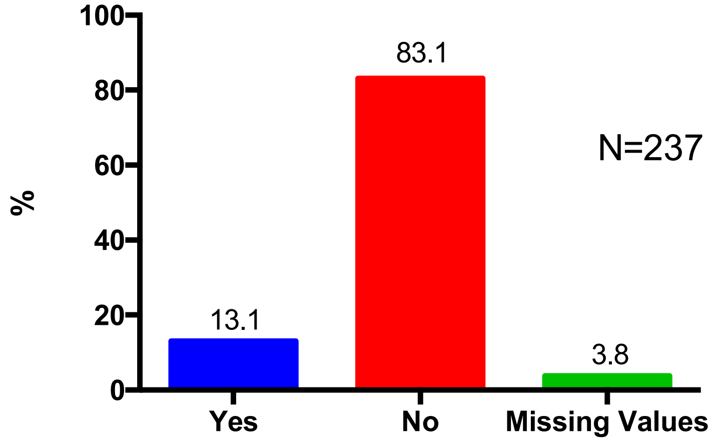

Figure S8. Other Medications for Mental Health Conditions

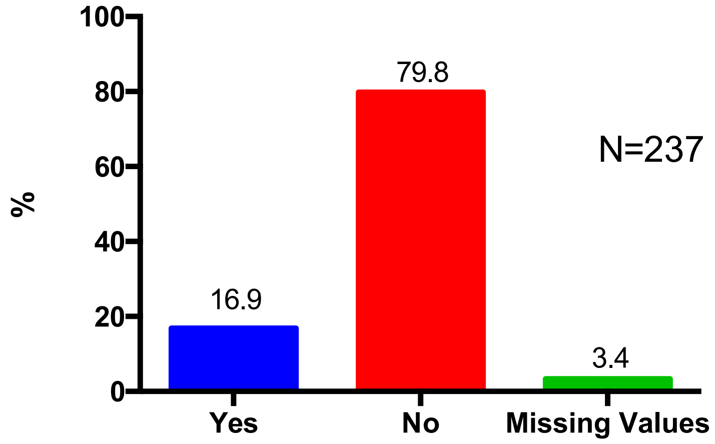

Figure S9. Warfarin or DOACS

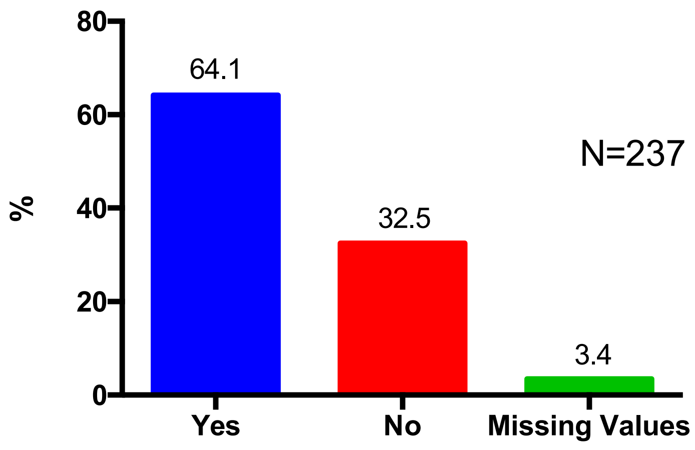

Figure S10. Hypertension

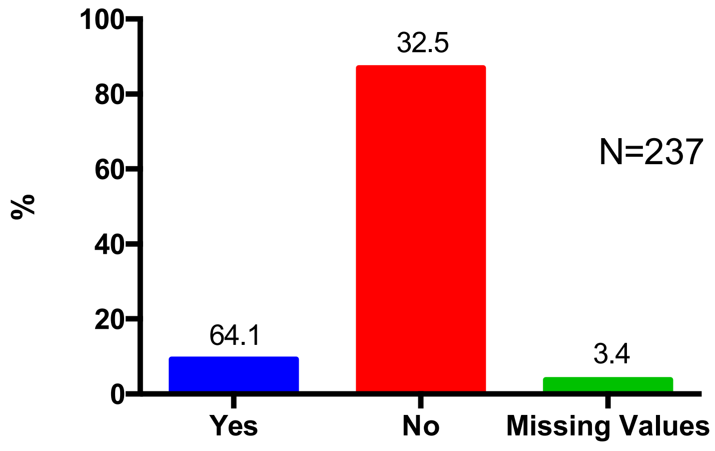

Figure S11. Congestive Heart Failure

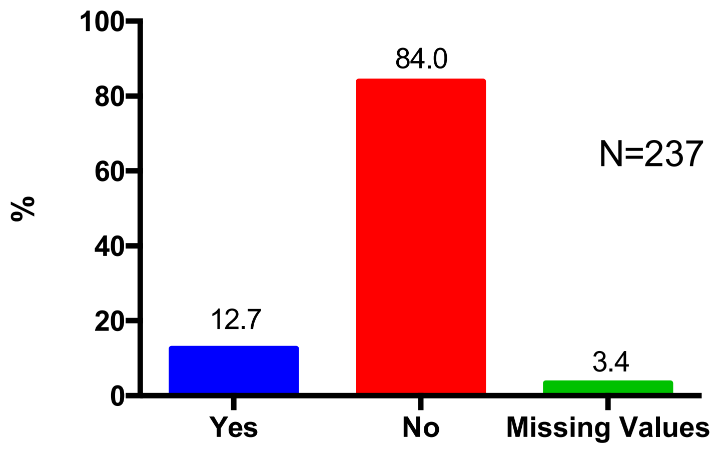

Figure S12. Asthma

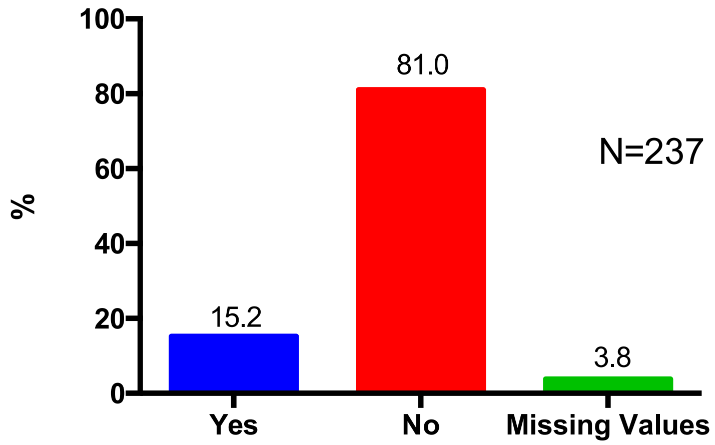

Figure S13. COPD

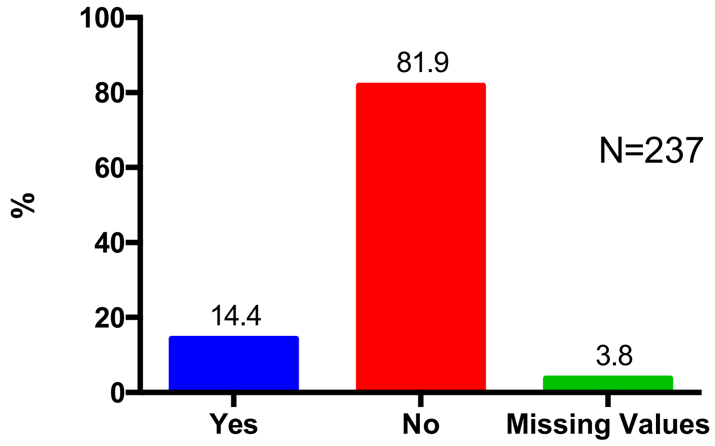

Figure S14. Osteoporosis

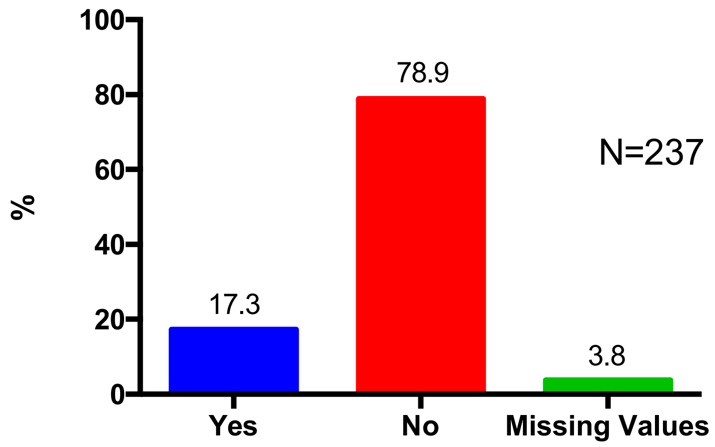

Figure S15. Fall in Previous Year

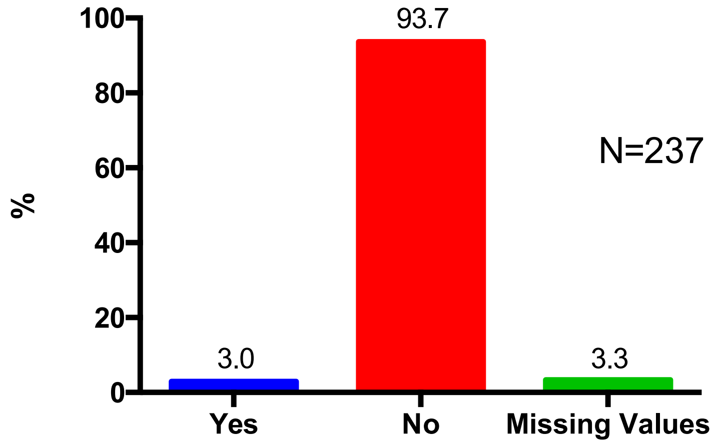

Figure S16. Active Cancer Treatment

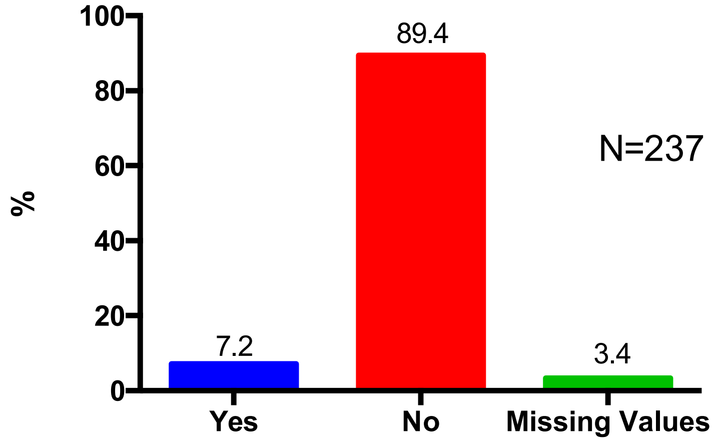

Figure S17. Cancer Survivor
